# Smoking habits in HIV-infected people compared with the general population in Italy: a cross-sectional study

**DOI:** 10.1186/s12889-020-08862-8

**Published:** 2020-05-20

**Authors:** Giuseppe Vittorio De Socio, Marta Pasqualini, Elena Ricci, Paolo Maggi, Giancarlo Orofino, Nicola Squillace, Barbara Menzaghi, Giordano Madeddu, Lucia Taramasso, Daniela Francisci, Paolo Bonfanti, Francesca Vichi, Marco dell’Omo, Luca Pieroni, On behalf of CISAI study group, Carmen Santoro, Tiziana Quirino, Maddalena Farinazzo, Federica Magne, Chiara Molteni, Lisa Malincarne, Erika Riguccini, Marco Nofri, Paola Bagella, Maria Sabrina Mameli, Beatrice Tiri, Marta Guastavigna

**Affiliations:** 1 Department of Medicine 2, Infectious Diseases Unit, Azienda Ospedaliera di Perugia and University of Perugia, Santa Maria Hospital, Perugia, Italy; 2 Current Address: Azienda Ospedaliera di Perugia, Piazzale Menghini 1, 06129 Perugia, Italy; 3 Department of Political Science, University of Perugia, Perugia, Italy; 4 Department of Woman, Newborn and Child, Fondazione IRCCS Ca’ Granda Ospedale Maggiore Policlinico, Milan, Italy; 5 Infectious Diseases Clinic, University of Campania “Luigi Vanvitelli”, Naples, Italy; 6 Division I Infectious and Tropical Diseases, ASL Città di Torino, Turin, Italy; 7 Infectious Diseases Unit ASST-MONZA, San Gerardo Hospital-University of Milano-Bicocca, Monza, Italy; 8 Infectious Diseases Unit, ASST della Valle Olona – Busto Arsizio (VA), Busto Arsizio, Italy; 9 Department of Medical, Surgical and Experimental Sciences, University of Sassari, Sassari, Italy; 10 Department of Health Science (DISSAL), Infectious Disease Clinic, University of Genova, Genoa, Italy; 11 Infectious Diseases Unit, Department of Internal Medicine, Fondazione IRCCS Ca’ Granda Ospedale Maggiore Policlinico, Milan, Italy; 12 Infectious Diseases Unit, A. Manzoni Hospital, Lecco, Italy; 13 Infectious Diseases Unit, Santa Maria Annunziata Hospital, USL Centro, Florence, Italy; 14 Department of Medicine, Perugia University, Perugia, Italy

**Keywords:** Smoking, Tobacco, Italy, Lifestyle, HIV, AIDS, Cardiovascular disease

## Abstract

**Background:**

Tobacco use is a leading cause of preventable diseases and death for all individuals, even more so for people living with HIV (PLWH), due to their status of chronic inflammation. To date, in Italy no study was performed to compare smoking habits in PLWH and the general population. We aimed to investigate smoking habits in PLWH, as compared to the general population.

**Methods:**

Multi-center cross-sectional study. Smoking habits were compared between PLWH and the general population. PLWH were enrolled in the STOPSHIV Study. The comparison group from the general population was derived from a survey performed by the National Statistics Institute (ISTAT), with a stratified random sampling procedure matching 2:1 general population subjects with PLWH by age class, sex, and macro-area of residence.

**Results:**

The total sample consisted of 1087 PLWH (age 47.9 ± 10.8 years, male 73.5%) and 2218 comparable subjects from the general population. Prevalence of current smokers was 51.6% vs 25.9% (*p* < 0.001); quitting smoking rate was 27.1% vs. 50.1% (*p* < 0.001) and the mean number of cigarettes smoked per day was 15.8 vs. 11.9 (*p* < 0.001), respectively for PLWH and the general population. Smoking and heavy smoking rates amongst PLWH were significantly higher even in subjects who reported diabetes, hypertension and extreme obesity (*p* < 0.001). Logistic regressions showed that PLWH were more likely current smokers (adjusted Odds Ratio, aOR = 3.11; 95% Confidence Interval (CI) =2.62–3.71; *p* < 0.001) and heavy smokers (> 20 cigarettes per day) (aOR = 4.84; 95% CI = 3.74–6.27; *p* < 0.001). PLWH were less likely to have quitted smoking (aOR = 0.36; 95% CI = 0.29–0.46; *p* < 0.001).

**Conclusion:**

HIV-infected patients showed a higher rate of current smokers, a larger number of cigarettes smoked and a lower quitting rate than the general population. Our findings emphasize the need for smoking cessation strategies targeting HIV persons.

## Background

Over the past two decades, HIV-associated morbidity and mortality rates have dramatically declined and the life expectancy of people living with HIV (PLWH) has considerably increased, due to effective antiretroviral therapy (ART). In Italy around 90% of PLWH starting HIV treatment achieve and maintain viral suppression [1, 2], so their life expectancy is near that of HIV-negative people their own age. The prolonged life expectancy of PLWH on ART led to significant changes in causes of death, with a shift from AIDS-related to non-AIDS-related causes, including cardiovascular disease, chronic obstructive lung disease (COPD), lung cancer and other non-AIDS-related neoplasms [3, 4]. Since cigarette smoking is a relevant risk factor for these diseases, and life expectancy for smokers is at least 10 years shorter than for nonsmokers [5], cigarette smoking is a major concern for PLWH. PLWH who receive optimal ART and other health care interventions appear to lose more life years due to tobacco smoking than to HIV infection [6]. Cigarette smoking increases morbidity and mortality for HIV related and non-HIV related diseases and contributes to an acceleration of the aging process [7]. Lung cancer, cardiovascular diseases, and COPD are major concerns [8, 9]. Thus, identifying and appropriately managing smokers with HIV infection is a relevant clinical issue and current medical guidelines for PLWH mandate preventive interventions for lifestyle modification to improve their prognosis [10].

Epidemiological studies performed in the USA and other industrialized countries have demonstrated that the prevalence of smokers among PLWH is two to three times higher than that of reference populations [11, 12] To date, no data comparing smoking habits between PLWH and the general population have been published with regards to Italy.

In this study, we present the comparison between two cross-sectional surveys. We examined the characteristics of smoking habits in a sample of PLWH from Italy, enrolled in a study on smoking prevalence and cessation (STOPSHIV study) [13, 14], and in a matched comparison sample from the Italian general population, selected from a survey performed by the National Statistics Institute (ISTAT) [15].

## Methods

This study was conducted by the Coordinamento Italiano per lo Studio di Allergie e Infezione da HIV (CISAI, Italian coordinating group for the study of allergies and HIV infection, www.cisai.it), a nationwide network of Italian Infectious Diseases Clinics. PLWH were enrolled in the STOPSHIV study (Stop Smoking in HIV infection), a prospective cohort study on smoking habits. A detailed description of the STOPSHIV study was previously published [13]. Briefly, all consecutive HIV positive patients, aged ≥18 years and attending scheduled or unscheduled outpatient visits at one of the CISAI group centers were invited to participate in the study from July 2014 through December 2016. The participation was complete. Smoking habits were investigated by a standardized questionnaire (available as supplementary material) and data regarding HIV patients such as anthropometric measures, blood pressure, and history of diabetes and co-morbidities were recorded using a standard data collection form as per previous studies by the CISAI study group [16]. The study was approved by the institutional ethics committee of the coordinating center (Ethics Committee of the Umbria Region, no. 3896/14 and 5964/15) and participating centers. All subjects provided written informed consent to participate in the study. Confidentiality was assured since all patient information was anonymized, and secured storage was prepared for both paper questionnaires and electronic dataset.

A comparison group of individuals from the general population without a verified HIV positive test was drawn through a stratified random sampling procedure, using a 2015 sample of the Italian population.

Specifically, data were drawn from the ISTAT annual sample survey “Aspetti della vita quotidiana” (“Aspects of daily life”), a cross-sectional nationally representative dataset covering the resident population in private households and involving more than 45,000 individuals (Pen-and-paper-interview technique). Information on gender, age group, ethnicity, region of residence, marital status, education, co-morbidities and smoking habits were collected by standardized questionnaire [15]. Co-morbidities considered for purposes of this study were hypertension, diabetes and severe obesity (body mass index, BMI ≥40 Kg/m^2^ according to the Centers for Disease Control and Prevention (CDC) [17]).

Since health-related variables were self-reported amongst the general population, it is likely that those subjects reporting hypertension or diabetes were being treated for these diagnoses. We harmonized data collection from STOPSHIV, classifying HIV patients as hypertensive or diabetics if they were treated for the condition when recruited.

The overall comparison group sample was selected through a stratified random sampling procedure. Overall, this technique consisted in dividing the entire general population into different strata based on some specific characteristics and then randomly select the final subjects proportionally from the prevalence of the selected characteristics in the comparison population. More specifically, it consisted of subjects randomly chosen from a representative sample of the Italian population, to match the PLWH sample 2:1. Matching variables were age, sex and macro-area of residence (North, Center and South/Islands).

Though PLWH and people from the general population were interviewed for different projects, their smoking habits were examined using the same structured questionnaire. Subjects were questioned if they had smoked at least 100 cigarettes in their lifetime [18]. If so, they were asked if they had been smoking every day or some days during the last year, in addition to how many cigarettes per day, on average. Using this information, they were classified as never, current or former smokers.

### Outcome variables

Smoking prevalence: percentage of current smokers in the overall sample. Specifically, current smokers were identified as those persons who reported smoking 100 cigarettes or more during their lifetime and were currently smoking every day or some days. On the other hand, never smokers were defined as persons who reported not smoking more than 100 cigarettes in their entire life [12].

Quitting rate: prevalence of former smokers among all smokers (former + current). Specifically, former smokers were defined as those who reported smoking at least 100 cigarettes during their lifetime but were not currently smoking.

Heavy smoking prevalence: percentage of those who currently smoke more than 20 cigarettes per day in the overall sample [19].

Frequency of smoking: self-reported number of cigarettes smoked per day.

All outcome variables were compared between PLWH and the general population (main comparison).

Control variables were gender, age, citizenship (Italian vs. other), educational level (college degree, high school graduation and elementary school or lower), marital status (single, married/in stable relationship, separated/divorced, widowed), macro-area of residence, and alcohol abuse (intake of ≥35 g/die alcohol). Information on co-morbidities was also incorporated into the model.

### Statistical analysis

Categorical variables were described as frequency (%, with 95% confidence interval, CI) and comparisons between PLWH and general population were performed using the Pearson’s or Mantel-Hanzsel chi-square test (as appropriate). Continuous variables were described as mean (standard deviation, SD) and compared using the analysis of variance.

Smoking, heavy smoking and quitting rates were calculated as percentage and 95% CI (Wilson score interval method).

Odds ratios (OR) and 95% CI for current smoking, heavy smoking, and quitting were calculated using the general population group as the reference. Potential confounders such as education, marital status, ethnicity, alcohol abuse and co-morbidities were included in the logistic regression equation to account for characteristics that were different between PLWH and the general population. We also included age class, gender and macro-area of residence.

Since the presence of missing values, if any, was less than 6%, we presented estimates from the main variable complete cases. To check for robustness, we planned to replicate the analysis by using dummies for missing values. Data analysis was conducted using the statistical software STATA 14.

## Results

Overall, 1087 HIV patients were enrolled in 10 participating clinical centers. In the PLWH group, mean age was 47.9 years (SD 10.8), and most subjects were on ART at study onset, with undetectable viral load.

The comparison group consisted of a sample of 2218 subjects matched for gender, age class and area of residence*.*

The main characteristics of study groups are reported in Table 1. Given the high prevalence of non-Caucasian subjects among PLWH, groups were significantly different with regards to ethnicity. They also differed in terms of marital status, education and co-morbidities.

**Table 1:** Characteristics of 1087 people living with HIV (PLWH) vs 2218 subjects from the Italian general population

	PLWH *N* = 1087 N (%)	General population *N* = 2218 N (%)	*p*-value
**Gender**			
Male	799 (73.5)	1623 (73.2)	0.84
**Age group**			
< 25	13 (1.2)	29 (1.3)	
25–34	115 (10.5)	235 (10.6)	
35–44	247 (22.7)	511 (23.0)	
45–54	454 (41.8)	916 (41.3)	
55–64	185 (17.0)	376 (17.0)	
> 65	73 (6.8)	151 (6.8)	NS
**Marital status**			
Single	466 (42.9)	578 (26.1)	
Stable relationship	426 (39.1)	1350 (60.9)	
Separated/Divorced	114 (10.5)	232 (10.4)	
Widowed	30 (2.7)	58 (2.6)	
*Missing*	*51 (4.7)*	–	< 0.001
**Region of residence**			
Northern Italy	346 (31.8)	709 (32.0)	
Central Italy	449 (41.3)	910 (41.0)	
Southern Italy	292 (26.9)	599 (27.0)	0.99
**Citizenship**			
Foreign	117 (10.8)	169 (7.6)	0.002
**Education**			
College degree	264 (24.5)	365 (16.4)	
High school graduation	519 (48.2)	948 (42.74)	
Elementary or lower	294 (27.2)	905 (40.8)	< 0.001
*Missing*	*10 (0.9)*	–	
**Chronic diseases**			
Diabetes	64 (5.8)	87 (3.9)	< 0.01
*Missing*	*22 (2.0)*	*96 (4.3)*	
Hypertension	199 (18.3)	304 (13.7)	< 0.001
*Missing*	*64 (5.8)*	*81 (3.6)*	
Extreme Obesity	20 (1.8)	228 (10.2)	< 0.001
*Missing*	*1 (0.1)*	–	
**Alcohol abuse**			
(35 g per day or more)	68 (3.1)	54 (4.9)	
*Missing*	–	*39 (3.6)*	< 0.01
**Smoking habits**	**% (CI) or mean (SD)**		
Current smokers	51.6 (48.6–54.6) *N* = 561	25.9 (24.0–27.7) *N* = 575	< 0.001
Former smokers	19.2 (16.9–21.6) *N* = 209	26.0 (24.1–27.8) *N* = 577	< 0.001
Never smokers	29.1 (26.4–31.8) *N* = 317	48.0 (45.9–50.1) *N* = 1066	< 0.0001
Number of cigarettes in current smokers	15.8 (SD 8.8)	12.0 (SD 6.1)	< 0.01
Heavy smokers	22.5 (20.0–25.0)	6.3 (5.3–7.4)	< 0.001
Quitting rate	27.1 (24.0–30.3)	50.1 (47.1–52.9)	< 0.001

Prevalence of current smokers was 51.6% (95% CI = 48.6–54.6) for PLWH and 25.9% (95% CI = 24.0–27.7) for the general population (Table 1). Among current smokers the mean number of cigarettes smoked was 15.8 (SD 8.8) in PLWH and 12.0 (SD 6.1) in the general population (all *p* < 0.001).

Across gender and age groups smoking habits were consistently different between PLWH and the general population, with a remarkably higher proportion of ever smokers in the STOPSHIV group. Among current smokers, the mean number of cigarettes per day was 10.2 (SD 5.9) in general population women vs. 14.3 (SD 8.1) in PLWH women, and 12.4 (SD 6.1) in general population men vs. 16.3 (SD 8.9) in PLWH men (both *p* < 0.001).

Overall, the quitting rate was: 27.1% (95% CI = 24.0–30.3) in PLWH and 50.1 (95% CI = 47.1–52.9) in the general population. Across gender and age groups the quitting rate in PLWH was significantly lower in any age group compared with the general population (*p* < 0.001) and in macro-area subgroups (data not shown). The quitting rate was lower in PLWH males aged 18–34 years (6.3, 95% CI = 1.5–12.5) compared with the general population (36.1, 95% CI = 26.8–45.5). The heavy smoking prevalence was significantly higher in PLWH (22.6%; 95% CI = 20.0–25.0) compared with the general population (6.3%; 95% CI = 5.3–7.4).

**Fig. 1:**
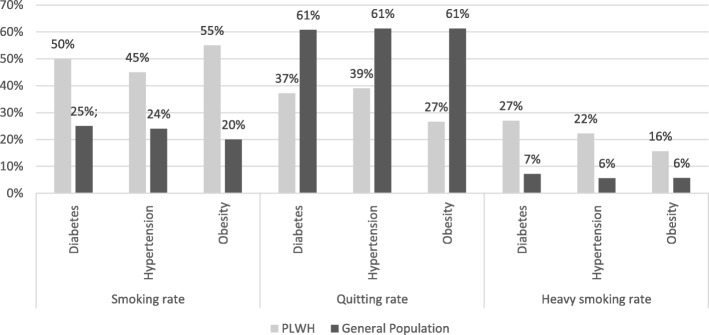
Outcomes by chronic diseases in people living with HIV (PLWH) and the Italian general population

Specifically, the smoking and heavy smoking rates amongst PLWH (Fig. 1) were significantly higher even in subgroups with high cardiovascular risk, i.e. those who reported diabetes, hypertension and extreme obesity (*p* < 0.001) (Supplemental material, Table 1S).

In PLWH, as compared to general population, crude ORs was 3.04 (95% CI 2.61–3.54) for current smoking, 4.30 (95% CI 3.44–5.38) for heavy smoking, and 0.37 (95% CI 0.30–0.45) for quitting (crude ORs for all variables in Supplemental material, Table 2S). In the adjusted analysis (Table 2), the findings of the fully adjusted logistic regression model were very similar. PLWH were significantly more likely to smoke compared with the general population group (adjusted OR, aOR = 3.11; 95% C.I. = 2.62–3.71; *p* < 0.001) and more than four times as likely to smoke greater than 20 cigarettes per day (aOR = 4.84; 95% C.I. = 3.74–6.27; *p* < 0.001). Moreover, our estimates show that PLWH were less likely to quit smoking (aOR = 0.36; 95% C. I = 0.29–0.46; *p* < 0.001) and that being a PLWH was associated with higher number of cigarettes smoked per day (on average, + 4 cigarettes per day). All analyses were also run including dummies for missing values, without significant modifications in the results.

**Table 2:** Adjusted Odds Ratios and corresponding 95% confidence intervals (CI) for smoking outcome measures of 1087 people living with HIV (PLWH) and 2218 subjects from the Italian general population

VARIABLES	Current smoking	Quitting	Heavy smoking
	OR (95% CI)	OR (95% CI)	OR (95% CI)
**Sex**			
**Male**	1.00	1.00	1.00
**Female**	0.64 ***	1.01	0.44 ***
	(0.53–0.77)	(0.78–1.31)	(0.31–0.6)
**Age group, years**			
< 25	0.63	0.59	0.29
	(0.3–1.29)	(0.15–2.26)	(0.06–1.3)
25–34	1.02	0.76	0.60 **
	(0.77–1.35)	(0.52–1.12)	(0.39–0.94)
35–44	0.74 ***	1.05	0.51 ****
	(0.6–0.92)	(0.79–1.4)	(0.36–0.72)
45–54	1.00	1.00	1.00
55–64	0.78 **	1.52 ***	0.87
	(0.62–0.98)	(1.15–2.00)	(0.63–1.2
> = 65	0.28 ***	3.82 ***	0.28 ***
	(0.18–0.43)	(2.42–6.05)	(0.14–0.54)
**Marital status**			
**Single**	1.00	1.00	1.00
Stable relationship	0.84 *	1.39 **	1.01
	(0.69–1.02)	(1.08–1.78)	(0.76–1.35)
Separated/divorced	1.18	1.11	1.37
	(0.88–1.56)	(0.77–1.59)	(0.9–2.08)
Widowed	1.08	1.3	2.7 ***
	(0.64–1.82)	(0.68–2.49)	(1.39–5.24)
**Education**			
College degree	1.00	1.00	1.00
High school graduation	1.09	1.01	1.61 ***
	(0.87–1.35)	(0.76–1.35)	(1.12–2.32)
Elementary or lower	1.37 ***	0.79	2.29 ***
	(1.09–1.73)	(0.58–1.07)	(1.57–3.34)
**Citizenship**			
Italian	1.00	1.00	1.00
Foreign	0.67 ***	0.51 ***	0.68
	(0.5–0.91)	(0.32–0.82)	(0.41–1.12)
**Comorbidity**			
No diabetes	1.00	1.00	1.00
Diabetes	1.14	0.95	1.49 *
	(0.76–1.7)	(0.59–1.53)	(0.88–2.5)
No hypertension	1.00	1.00	1.00
Hypertension	0.9	1.28 *	0.84
	(0.7–1.15)	(0.95–1.71)	(0.59–1.19)
No severe obesity	1.00	1.00	1.00
Severe obesity	0.65 **	1.61 **	0.69
	(0.46–0.92)	(1.06–2.44)	(0.38–1.26)
No alcohol abuse	1.00	1.00	1.00
Alcohol Abuse	0.89	0.97	0.97
	(0.58–1.36)	(0.54–1.71)	(0.53–1.77)
**Region**			
Northern Italy	1.00	1.00	1.00
Central Italy	0.89	1.20	0.87
	(0.73–1.07)	(0.95–1.53)	(0.64–1.18)
Southern Italy	1.15	0.58 ***	1.67 ***
	(0.93–1.41)	(0.44–0.77)	(1.23–2.25)
**Group**			
General population	1.00	1.00	1.00
**PLWH**	3.11 ***	0.36 ***	4.84 ***
	(2.62–3.71)	(0.29–0.46)	(3.74–6.27)

Note: *OR* Odds ratio; *CI* Confidence interval; Estimates were controlled for the entire set of covariates (age, gender, ethnic origin, educational level, marital status, region of residence, alcohol abuse, hypertension, diabetes, and extreme obesity). Estimates were performed by pooling together the PLWH and the general population samples

* *p* < 0.05; ** *p* < 0.01; ***p < 0.001

Factors affecting smoking habits were also sex, since women were less likely to be both current and heavy smokers; marital status, with subjects in a stable relationship being less likely current smokers and more likely former smokers; education, with an inverse relation with both current and heavy smoking; ethnicity, since non-Italian native subjects were less likely current smokers. Among co-morbidities, severe obesity was associated with quitting rate.

In order to explore any potential collider bias due to a possible reverse association between comorbidities and both smoking and HIV, we performed a stratified analysis (Supplemental material, Tables 3S, 4S and 5S). Results on the association between comorbidities and smoking outcomes stratified by study show similar non statistically significant odds ratios in PLWH and in the general population, except for extreme obesity. More specifically, our results suggest that extreme obesity in the general population is significantly inversely associated to smoking and positively associated to quitting, whereas extreme obesity was associated with lower, not significant, quitting rate in PLWH. However, since the sample size of extreme obese PLWH was very small, results should be taken with caution, suggesting, at least, a possible underestimation of main results. Finally, the association between HIV and smoking outcomes does not significantly vary in subgroups with and without comorbidities (Supplemental material Tables 6S, 7S and 8S).

## Discussion

The main results of this investigation revealed that HIV-infected patients who refer to our network of Infectious Disease Clinics for routine outpatient clinical care demonstrate levels of tobacco smoking remarkably higher than a matched sample from the general population: the prevalence of current smoking was higher, and among current smokers the mean number of cigarettes smoked daily was greater.

These Italian data are consistent with previously published research on PLWH from other countries: a recent meta-analysis [20] reported that the pooled prevalence of current smoking among women was 36.3% (95% CI 28.0–45.4%) and 50.3% among men (95% CI 44.4–56.2%); the quitting rate was twice as high in the general population than in PLWH group. In comparison with a study from a USA population [12] we found that quitting rate was similar in the general population group (50.1% vs. 51.7% in the USA general population) but lower in PLWH (27.1% vs. 32.4% in the USA).

It is reasonable to hypothesize that the negative health effects of tobacco smoking, an already alarming and widespread problem in the Italian general population [21], may be even more deleterious in PLWH. Over the last two decades, ART improvements have gradually prolonged life expectancy in PLWH; at the same time, cigarette smoking has emerged as the most important health hazard, as it is widespread among these patients and increases their morbidity and mortality.

Since PLWH are at high cardiovascular risk, the management of modifiable behavioral risk factors is a relevant issue. In this setting, smoking habits are really a key factor, considering that PLWH still tend to be undertreated for cardiovascular prevention [22] and that tailored interventions for smoking cessation have been proven effective in this population [15, 23]. Indeed, HIV-positive patients smoke more than the general population and demonstrate a lower quitting rate despite being at higher risk for cardiovascular diseases [11, 12].

In our study, we found that among ever smokers about a 25% of PLWH and more than half the general population had stopped smoking at the time of data collection. This low quitting rate among PLWH may be related to many factors, such as a high level of nicotine dependence and/or with a faster nicotine metabolism observed in PLWH [24]. In our study, we could not directly compare the degree of nicotine dependence between PLWH and the general population, because this information was not collected in the ISTAT survey. However, PLWH demonstrated a higher daily smoking frequency and prevalence of subjects smoking more than 20 cigarettes a day. Since daily cigarette consumption is one of the main determinants of the individual level of nicotine addiction, as assessed by the Heaviness of smoking index [25] it is conceivable that the nicotine dependence degree was also higher in PLWH than in general population.

Further in-depth research should investigate principal causes of the higher smoking prevalence among PLWH, in order to better identify the role of specific risk behaviors. The perceived lower life expectancy could likely lead to lower rates of quitting and reduced smoking [26]. Indeed, a better identification of underlying causes could be useful in implementing preventive strategies. In clinical practice, a possible barrier hindering anti-smoking strategies may be related to time constraints. In this setting, smoking as a health issue is considered a lower priority due to the reduced amount of time available in PLWH [27]. On the contrary, HIV specialists should be given the tools to address the increasingly complex demands of comprehensive HIV care, considering the rising importance of HIV-associated non-AIDS conditions in the aging HIV population [28].

Finally, smoking has a known detrimental impact on physical health and on the economic burden through avoidable healthcare expenditures [29, 30]. The treatment of chronic diseases attributable to tobacco smoking is deemed to increase health care expenditures, because in Italy PLWH currently have a high life expectancy, thereby increasing the costs of treatment for smoking-related diseases for the HIV-infected population. Intervention for smoking cessation may be a cost-effective method to lower expenditures due to tobacco-related illnesses while simultaneously improving the quality of life of PLWH. Key steps for planning interventions are quantifying the size of the target population and identifying adequate anti-smoking strategies.

In this study, several limitations merit discussion. In the STOPSHIV study the number of HIV patients older than 65 years was low. We had no laboratory markers of smoking status, and the information about patients’ smoking status was self-reported. However, in other investigations the smoker activity and cessation levels corresponded well with measured exhaled CO levels, and numerous studies on this topic utilized self-reporting [31]. In addition, a further concern is the impossibility in evaluating the degree of nicotine dependence among the general population.

Another limitation was that the matched sample from the general population was still different from the PLWH group in terms of education, marital status, ethnicity and comorbidities. Moreover, substances abuse and sexual orientation have previously been associated with higher smoking. Since this information was not collected in the ISTAT survey, we could not account for these variables in the general population. However, any proportions of subjects, in the general population, with past or present drug dependence, or with non-heterosexual orientation, should tend to diminish the difference between PLWH and general population. Surprisingly, no significant result was found when we analyzed the association between alcohol abuse and smoking habits, maybe because of the low number of subjects with this kind of abuse both in PLWH and the general population.

This study also has several strengths. We examined a well-characterized and unselected multicenter sample of Italian HIV-infected outpatients treated at Infectious Disease Clinics, which likely represents all PLWH living in the study area, even though it does not formally represent the whole Italian HIV population. In fact, all consecutive HIV patients attending scheduled or unscheduled outpatient visits in the recruitment period were proposed participation into the study and no one refused, so selection was unlikely to occur. We adopted a validated and standardized procedure for all observed patients. We used 2015 ISTAT data as a comparator and accurately matched for the main characteristics of our sample population. Data collection was similar as regards demographic and smoking habits information, recorded by interview. The estimate smoking prevalence of 25.9% among the general population is consistent and not less to other investigation from Italy in which the overall prevalence was 21.4% [32]. Moreover, we were able to compare selected clinically relevant chronic conditions such as diabetes, hypertension and obesity. As regards comorbidities, we tried to harmonize the information from STOPSHIV and ISTAT survey by classifying PLWH as hypertensive or diabetic if they were on treatment for such conditions.

## Conclusions

HIV-infected patients showed a higher rate of current smokers, a larger number of cigarettes smoked and a lower quitting rate than the general population. Our findings emphasize the need for smoking cessation strategies targeting HIV persons.

## Supplementary information

**Additional file 1.** STOPSHIV questionnaire

**Additional file 2: Table S1.** Prevalence of comorbidities in strata of smoking habits. **Table S2.** Crude Odds Ratios and corresponding 95% confidence intervals (CI) for smoking outcome measures of 1087 people living with HIV (PLWH) and 2218 subjects from the Italian general population. **Table S3.** Fully adjusted association between hypertension and smoking habits in strata of study (no hypertension is the reference category). **Table S4.** Fully adjusted association between diabetes and smoking habits in strata of study (no diabetes is the reference category). **Table S5.** Fully adjusted association between extreme obesity and smoking habits in strata of study (no extreme obesity is the reference category). **Table S6.** Fully adjusted association between study population and smoking habits in strata of hypertension (general population is the reference category). **Table S7.** Fully adjusted association between study population and smoking habits in strata of diabetes (general population is the reference category). **Table S8.** Fully adjusted association between study population and smoking habits in strata of extreme obesity (general population is the reference category).

## Data Availability

Data can be made available from the corresponding author on reasonable request.

## Abbreviations

PLWH: People living with HIV
ART: Antiretroviral therapy
COPD: Chronic obstructive lung disease
ISTAT: National statistics institute
CISAI, Italian coordinating group for the study of allergies and HIV infection: Coordinamento Italiano per lo Studio di Allergie e Infezione da HIV
STOPSHIV: Stop Smoking in HIV infection study
CI: Confidence interval
SD: Standard deviation
OR: Odds ratio
aOR: Adjusted odds ratio
BMI: Body mass index
CDC: Centers for disease control and prevention
