# Supplementary material for: Smoking habits in HIV-infected people compared with the general population in Italy: a cross-sectional study
Source: BMC Public Health. 2020 May 20;20:734. doi: 10.1186/s12889-020-08862-8 (PMC7238525; doi:10.1186/s12889-020-08862-8)
Supplement: Supplementary file 1 — Additional file 1. STOPSHIV questionnaire [file 12889_2020_8862_MOESM1_ESM.docx]

| 1. **5** | In your entire life, did you smoke al least 100 cigarettes (5 packs)? | |  | | | no □ | | yes □ | |
| --- | --- | --- | --- | --- | --- | --- | --- | --- | --- |
|  | ***If yes, go on*** | | | | | | | | |
| 1. **6** | How old were you when you first started to smoke cigarettes regularly? | | .......... years | | | | | | |
|  | During the past year on how many days did you smoke cigarettes? | | - Never □  - Most days □  - Every day □ | | | | | | |
|  | ***If never, go to question 17*** | | | |  | | | |  |
| 1. **8** | During the past year, how many cigarettes a day did you smoke, on average? | | On average, I smoked .......... cigarettes a day | | | | | | |
| 1. **9** | How soon after you wake up do you smoke your first cigarette? | | - within 5 minutes  - 6-30 minutes  - 31-60 minutes  - more than 60 minutes | | | □  □  □  □ | |  | |
|  | Do you find it difficult to refrain from smoking in places where it is forbidden? | | |  | | no □ | | yes □ | |
| 1. **9** | Which cigarette would you hate most to give up? | | - the first cigarette in the morning  - any other | | | | □  □ |  | |
| 1. **10** | Do you smoke more frequently during the first hours after waking than during the rest of the day? | | |  | | no □ | | yes □ | |
| 1. **10** | Do you smoke even if you are so ill that you are in bed most of the day? | | |  | | no □ | | yes □ | |
| 1. **10** | Since you began to smoke regularly, did you ever quit for at least six months? | | |  | | no □ | | yes □ | |
| 1. **15** | Are you thinking of quitting smoking in the next 6 months? | | |  | | no □ | | yes □ | |
| 1. **16** | Are you thinking of quitting smoking in the next month? | | |  | | no □ | | yes □ | |
| 1. **18** | On a scale from one to ten, how much are you interested in quitting smoking?  **0** 1 2 3 4 5 6 7 8 9 **10**  ***Not at all Very interested*** | | | | | | | | |
| 1. **18** | On a scale from one to ten, how much do you think you are capable of quitting smoking?  **0** 1 2 3 4 5 6 7 8 9 **10**  ***Not at all Very capable*** | | | | | | | | |
| 1. **19** | How much do you think your smoking habits affect HIV infection progression? | Not at all  Not much  Pretty much  Very much | | | | □  □  □  □ | |  | |
| 1. **20** | How much do you think your smoking habits affect your future health? | Not at all  Not much  Pretty much  Very much | | | | □  □  □  □ | |  | |

| 1. **21** | How old were you when you quitted smoking? | I was ......... years old |
| --- | --- | --- |
| 1. **22** | During the year before quitting smoking, how many cigarettes did you smoke a day, on average? | On average, I smoked.......... cigarette a day |
