# Supplementary material for: Smoking habits in HIV-infected people compared with the general population in Italy: a cross-sectional study
Source: BMC Public Health. 2020 May 20;20:734. doi: 10.1186/s12889-020-08862-8 (PMC7238525; doi:10.1186/s12889-020-08862-8)
Supplement: Supplementary file 2 — Additional file 2: Table S1. Prevalence of comorbidities in strata of smoking habits. Table S2. Crude Odds Ratios and corresponding 95% confidence intervals (CI) for smoking outcome measures of 1087 people living with HIV (PLWH) and 2218 subjects from the Italian general population. Table S3. Fully adjusted association between hypertension and smoking habits in strata of study (no hypertension is the reference category). Table S4. Fully adjusted association between diabetes and smoking habits in strata of study (no diabetes is the reference category). Table S5. Fully adjusted association between extreme obesity and smoking habits in strata of study (no extreme obesity is the reference category). Table S6. Fully adjusted association between study population and smoking habits in strata of hypertension (general population is the reference category). Table S7. Fully adjusted association between study population and smoking habits in strata of diabetes (general population is the reference category). Table S8. Fully adjusted association between study population and smoking habits in strata of extreme obesity (general population is the reference category). [file 12889_2020_8862_MOESM2_ESM.docx]

**Supplemental material**

**Table 1S.** Prevalence of comorbidities in strata of smoking habits.

| Variables | | PLWH | General Population |
| --- | --- | --- | --- |
|  |  | N (%) | N (%) |
| Smoking rate | Diabetes | 32/64(50) | 22/87 (25) |
|  | Hypertension | 89/199 (45) | 73/304 (24) |
|  | Obesity | 11/20 (55) | 46/228 (20) |
| Quitting rate | Diabetes | 19/51 (37) | 34/56 (61) |
|  | Hypertension | 57/146 (39) | 116/ 189 (61) |
|  | Obesity | 4/15 (27) | 73/119 (61) |
| Heavy smoking rate | Diabetes | 19/64 (27) | 6/87 (7) |
|  | Hypertension | 43/199 (22) | 17/304 (6) |
|  | Obesity | 3/20 (16) | 13/228 (6) |

Note: The quitting rate is defined as the prevalence of former smokers among all smokers (former + current), heavy smoking rate as the prevalence of heavy smokers (>20 cigarettes/day) among current smokers.

**Table 2S.** Crude Odds Ratios and corresponding 95% confidence intervals (CI) for smoking outcome measures of 1087 people living with HIV (PLWH) and 2218 subjects from the Italian general population.

|  | **Current smoking** | **Quitting** | **Heavy smoking** |
| --- | --- | --- | --- |
|  | **Crude OR (95% CI)** | **Crude OR (95% CI)** | **Crude OR (95% CI)** |
| **Sex** (ref. M) |  |  |  |
| **F** | 0.67 *** | 0.85 | 0.53 *** |
|  | (0.57 – 0.80) | (0.68 – 1.07) | (0.40 – 0.70) |
| **Age group, years (ref. 45-54)** |  |  |  |
| < 25 | 0.63 | 0.42 | 0.27 * |
|  | (0.32 – 1.24) | (0.11 – 0.51) | (0.06 – 1.14) |
| 25-34 | 1.04 | 0.67 * | 0.55 ** |
|  | (0.81 – 1.32) | (0.47 – 0.94) | (0.37 – 0.82) |
| 35-44 | 0.71 *** | 0.97 | 0.44 *** |
|  | (0.59 – 0.86) | (0.75 – 1.25) | (0.32 – 0.60) |
| 55-64 | 0.77 *** | 1.66 *** | 0.85 |
|  | (0.62 – 0.94) | (1.30 – 2.12) | (0.64 – 1.13) |
| >=65 | 0.28 *** | 4.93 *** | 0.28 *** |
|  | (0.19 – 0.41) | (3.26 – 7.47) | (0.15 – 0.53) |
| **Marital status** (ref. single) |  |  |  |
| Stable relationship | 0.63 *** | 1.98 *** | 0.77 *** |
|  | (0.53 – 0.73) | (1.60 – 2.45) | (0.61 – 0.98) |
| Separated/divorced | 0.93 | 1.45 * | 1.06 |
|  | (0.72 – 1.20) | (1.05 – 2.00) | (0.72 – 1.52) |
| Widowed | 0.69 | 1.88 * | 1.40 |
|  | (0.43 – 1.11) | (1.06 – 3.33) | (0.78 – 2.52) |
| **Education** (ref. college degree) |  |  |  |
| High school graduation | 1.03 | 1.03 | 1.32 * |
|  | (0.85 – 1.26) | (0.80 – 1.32) | (0.96 – 1.81) |
| Elementary or lower | 1.00 | 1.11 | 1.51 * |
|  | (0.82 – 1.23) | (0.86 – 1.45) | (1.09 – 2.08) |
|  |  |  |  |
| **Citizenship** (ref. Italian) |  |  |  |
| Foreign | 0.74 *** | 0.52 *** | 0.57 * |
|  | (0.57 – 0.97) | (0.34 – 0.80) | (0.36 – 0.91) |
| **Comorbidity** (ref. N) |  |  |  |
| Diabetes | 1.04 | 1.48 * | 1.54 * |
|  | (0.74 – 1.47) | (1.00 – 2.19) | (0.99 – 2.41) |
| Hypertension | 0.87 | 1.74 *** | 1.03 |
|  | (0.71 – 1.07) | (1.37 – 2.20) | (0.76 – 1.38) |
| Severe obesity | 0.54 *** | 2.05 *** | 0.50 *** |
|  | (0.40 – 0.73) | (1.44 – 2.93) | (0.30 – 0.84) |
|  |  |  |  |
| **Alcohol Abuse** (ref. N) | 1.09 | 0.73 | 1.27 |
|  | (0.74 – 1.59) | (0.44 – 1.23) | (0.75 – 2.16) |
| **Region** (ref. Northern Italy) |  |  |  |
| Central Italy | 0.88 | 1.16 | 0.91 |
|  | (0.74 – 1.04) | (0.94 – 1.43) | (0.69 – 1.19) |
| Southern Italy | 1.16 | 0.61 *** | 1.62 *** |
|  | (0.96 – 1.40) | (0.48 – 0.79) | (1.23 – 2.11) |
| **Group (ref. general population)** |  |  |  |
| **PLWH** | 3.04 *** | 0.37 *** | 4.30 *** |
|  | (2.61 - 3.54) | (0.30 - 0.45) | (3.44 - 5.38) |

Note: OR, Odds ratio; CI, confidence interval

* p<0.05; ** p<0.01; ***p<0.001.

Estimates were performed by pooling together the PLWH and the general population samples.

**Table 3S.** Fully adjusted association between hypertension and smoking habits in strata of study (no hypertension is the reference category).

|  | Smoking | Quitting | Heavy smoking |
| --- | --- | --- | --- |
| In PLWH | 0.83 (0.58 - 1.2) | 1.31 (0.82 - 2.08) | 0.90 (0.58 - 1.39) |
| In general population | 1.05 (0.75 - 1.45) | 1.17 (0.80 - 1.71) | 0.83 (0.45 - 1.52) |

**Table 4S.** Fully adjusted association between diabetes and smoking habits in strata of study (no diabetes is the reference category).

|  | Smoking | Quitting | Heavy smoking |
| --- | --- | --- | --- |
| In PLWH | 1.12 (0.62 - 2.02) | 0.89 (0.43 - 1.83) | 1.58 (0.84 - 2.99) |
| In general population | 1.13 (0.65 - 1.97) | 0.95 (0.50 - 1.82) | 1.24 (0.46 - 3.32) |

**Table 5S.** Fully adjusted association between extreme obesity and smoking habits in strata of study (no extreme obesity is the reference category).

|  | Smoking | Quitting | Heavy smoking |
| --- | --- | --- | --- |
| In PLWH | 0.98 (0.27 - 3.48) | 0.37 (0.03 - 3.57) | 0.63 (0.07 - 5.41) |
| In general population | 0.59 ***  (0.41 - 0.86) | 1.72 * (1.12 - 2.64) | 0.72 (0.38 - 1.36) |

**Table 6S**. Fully adjusted association between study population and smoking habits in strata of hypertension (general population is the reference category).

|  | Smoking | Quitting | Heavy smoking |
| --- | --- | --- | --- |
| In hypertensive PLWH | 2.31 ***  (1.46 – 3.65) | 0.53 ***  (0.30 – 0.91) | 5.15 ***  (2.48 – 10.71) |
| In non-hypertensive PLWH | 3.30 ***  (2.73 – 3.99) | 0.33 ***  (0.25 – 0.43) | 4.87 ***  (3.68 - 6.43) |

**Table 7S.** Fully adjusted association between study population and smoking habits in strata of diabetes (general population is the reference category).

|  | Smoking | Quitting | Heavy smoking |
| --- | --- | --- | --- |
| In diabetic PLWH | 2.74 ***  (1.15 – 6.49) | 0.27 ***  (0.07 – 0.94) | 11.34 ***  (2.81 – 45.87) |
| In non- diabetic PLWH | 3.12 ***  (2.61 – 3.72) | 0.36 ***  (0.28 – 0.46) | 4.77 ***  (3.66 – 6.22) |

**Table 8S.** Fully adjusted association between study population and smoking habits in strata of extreme obesity (general population is the reference category).

|  | Smoking | Quitting | Heavy smoking |
| --- | --- | --- | --- |
| In obese PLWH | 7.63 **  (1.43 – 40.67) | 0.08 **  (0.00 – 0.94) | 2.81 *  (0.23 – 0.33) |
| In non-obese PLWH | 3.08 ***  (2.58 – 3.67) | 0.37 ***  (0.29 – 0.47) | 4.84 ***  (3.73 – 6.28) |
